# Editorial: Comprehensive evaluation of various training protocols for youth: effects on body composition, hemodynamics, and motor performance 

**DOI:** 10.3389/fphys.2025.1644031

**Published:** 2025-07-03

**Authors:** Cristian Alvarez, Jarosław Domaradzki

**Affiliations:** ^1^ Exercise and Rehabilitation Sciences Institute, School of Physical Therapy, Faculty of Rehabilitation Sciences, Universidad Andres Bello, Santiago, Chile; ^2^ Faculty of Physical Education and Sport, Wroclaw University of Health and Sport Sciences, Wroclaw, Poland

**Keywords:** cardiometabolic disease, high-intensity interval training (HIIT), young, adults, human performance, moderate-intensity aerobic exercise

## 1 Introduction

There is a worrying increase in worsening cardiometabolic health conditions, including arterial hypertension (Organization, 2025) and diabetes (WHO, 2024), in several countries; different exercise training modalities, including moderate-intensity continuous (MICT), high-intensity interval (HIIT), resistance (RT), and concurrent training (CT), are widely recommended as preventive measures and treatment tools (Kanaley et al., 2022; Bull et al., 2020; Pescatello et al., 2019). In both young (i.e., children and adolescents in school environments) and adult populations, there is a need to increase the amount of exercise performed for these groups, as there is strong evidence for exercise treating these abnormalities; when performance is high, other specific exercise strategies are useful to optimize performance. In the present Research Topic, we aimed to summarize current exercise strategies for youth and adults at cardiometabolic risk or for performance optimization.

## 2 Short school-based interventions are effective in improving physical fitness and motor performance

Domaradzki et al. have, in their PEER-HEART study, examined (n = 307) adolescents to test two sets of 8-weeks of traditional HIIT and a plyometric HIIT (HIPT) in physical education classes. Both groups saw significantly reduced total body fat percentage and systolic/diastolic blood pressure (SBP/DBP) while increasing maximum oxygen uptake (VO2max). The authors conclude that brief HIIT or HIPT sessions are feasible for improving cardiovascular health in adolescents.

Jovanović et al. analyzed a 12-week school-based Tabata HIIT warm-up routine (2×/week, 4-min bouts), which replaced standard physical education class warm-ups, for (n = 30) Serbian boys (16 years), with 30 controls. Shuttle-run distance and estimated VO2max increased in both groups, but improvements were ∼1.4 fold larger with HIIT. HIIT additionally boosted standing long jump and countermovement jump performance and right handgrip strength (HGS) versus control. The authors conclude that brief Tabata HIIT is an efficient way to enhance cardiorespiratory fitness and lower limb power in adolescent boys.

Sun et al. have examined an 8-week randomized trial dividing (n = 18) sedentary, normal weight Chinese adolescents into thrice-weekly HIIT, MICT, or control groups. Both exercise modalities similarly reduced body fat mass and visceral fat area versus baseline. Only HIIT lowered waist-to-hip ratio and elicited marked falls in SBP (−6) and DBP (−11 mmHg) as well as triglycerides (−30%). The authors conclude that short, school-friendly HIIT is a more effective and time-efficient prescription than volume-matched MICT for reducing adiposity and cardiometabolic risk in sedentary youth.

## 3 The effects of different exercise modalities on body composition and hemodynamic parameters in youth

Min-Seong Ha studied a 16-week after-school combined exercise program (sports games plus MICT and RT), which enrolled (n = 33) Korean boys (11–12 years) with (n = 16) and without obesity (n = 17). In the obesity group, body fat percentage was reduced from 37.6% to 29.1% and muscle mass increased by ∼31%. C-peptide and resistin fell markedly (−1.6 and −3.0 ng mL^−1^), while insulin growth factor (IGF-1) and growth hormone increased by ∼20%–25%. C-peptide and IGF-1 were thus considered mechanistic markers of training responsiveness in adolescent boys with obesity.

Nowak et al. have carried out a cross-sectional analysis of (n = 495) male academy footballers (12–16 years) producing normative percentile charts for speed, endurance, and power tests. Running times over 5 m, 10 m, and 30 m, standing long-jump distance, and maximal aerobic speed (MAS) from the 30–15 intermittent fitness test were recorded during training blocks from 2018 to 2022. The sharpest gains appeared between ages 13 and 14: sprint times improved by 0.087–0.215 s (5–10 m) and 0.438–0.719 s (30 m), long-jump length rose 31–48 cm, and MAS increased 0.3–0.6 m s^−1^. Percentile grids (P3–P97) allow coaches to benchmark individual progress and detect outliers in development trajectories. These charts provide a practical tool for tailoring youth-soccer conditioning, optimizing load, and mitigating injury risk throughout adolescence.

Amare et al. conducted a randomized 8-week trial assigning (n = 24) inactive, overweight/obese Ethiopian men (∼49 years) to MICT, RT, or CT. All groups saw lowered fasting blood glucose, insulin resistance, SBP/DBP, and waist-to-hip ratio compared to their baselines. RT and CT produced larger fasting glucose declines than MICT, and insulin resistance was reduced with RT compared with MICT. SBP dropped most in CT and RT. The exercise modality explained up to 57% of the variance, summarizing that short-term RT or CT are effective exercise modalities for cardiometabolic risk reduction in overweight and obese middle-aged men.

## 4 The physiological mechanisms underlying exercise responses and support strategies

Feige et al. examined (n = 11) elite fin swimmers (children <17 years and adults 17–29 years) during dynamic apnea dives of 25–100 m. Transcutaneous pulse oximetry and heart-rate monitoring during repeated pool dives showed a stereotypical diving response-bradycardia during immersion followed by tachycardic rebound at surfacing. Longer apnea duration, rather than swim speed, produced the greatest falls in oxygen saturation, whereas higher speeds chiefly intensified cardiovascular workload. The authors conclude that real-life dynamic apnea evokes age-dependent cardiorespiratory stress and provide benchmark data to guide training and risk assessment in pediatric and adult divers.

Wu et al. studied (n = 90) male college athletes who completed 7 days of moderate-intensity training and were randomized to true transcranial pulse current stimulation (tPCS) or control. Daily 20-min tPCS at 1.5 mA was delivered immediately post-exercise, and fatigue was reported by subjective fatigue scale (RPE), functional near-infrared spectroscopy (fNIRS) cerebral oxygenation, and blood biomarkers. Compared with controls, the tPCS group reported lower RPE scores and showed smaller post-exercise drops in oxygenated hemoglobin concentration (Oxy Hb) and rises in deoxyhemoglobin concentration (HHb), total hemoglobin concentration (HbTot), and hemoglobin concentration difference (HbDiff). The authors conclude that brief daily tPCS is an effective, non-invasive countermeasure against the accumulation of exercise-induced fatigue by preserving central neural function.

Zhuan et al. applied a randomized crossover study testing whether adding three blood-flow-restriction (BFR) modes (continuous low, intermittent medium, and intermittent high to high-intensity [75% 1RM]) squat sessions boost lower limb and core muscle activation. Twelve RT college men performed three sets of eight deep squats under each BFR condition and a no-BFR control while electromyography, thigh circumference, and RPE were recorded. All BFR modes elevated vastus lateralis and vastus medialis maximum voluntary contraction during the first two sets versus the control. The authors conclude that continuous low-pressure BFR offers the most stable posterior-thigh engagement, while intermittent high-pressure BFR optimizes spinal-extensor activation and perceived exertion, making both viable add-ons to heavy squat training.

## 5 Conclusion and future directions

Taken together, all these studies support the concept that different exercise training modalities such as MICT, HIIT, RT, CT or other variations of these, increase the cardiorespiratory fitness and modify positively the body composition of young populations in school environments, dynamic apnea reduces diving risk, tPCS is effective in decreasing exercise-induced fatigue, and low-pressure BFR modes improve heavy squat training performance.

Nevertheless, several open Research Topic remain:

• There is a pressing need to explore inter-individual variability in exercise response and identify predictors of responsiveness in the context of responders and non-responders to the exercise stimuli.
• Future work should compare different durations, intensities, and modalities of training in diverse populations to identify the optimal dose for effective response in various morphological and physiological features.
• Long-term follow-ups are needed to assess whether effects observed in postintervention measurements translate into adult health benefits over long-term periods.
• Personalized and context-specific interventions (e.g., biological, social-economic, etc.) are needed to study the various conditions, diversity, and their effects.
